# Role of Majorana fermions in high-harmonic generation from Kitaev chain

**DOI:** 10.1038/s41598-022-10465-9

**Published:** 2022-04-25

**Authors:** Adhip Pattanayak, Sumiran Pujari, Gopal Dixit

**Affiliations:** Department of Physics, Indian Institute of Technology Bombay, Powai, Mumbai 400076 India

**Keywords:** High-harmonic generation, Topological matter

## Abstract

The observation of Majorana fermions as collective excitations in condensed-matter systems is an ongoing quest, and several state-of-the-art experiments have been performed in the last decade. As a potential avenue in this direction, we simulate the high-harmonic spectrum of Kitaev’s superconducting chain model that hosts Majorana edge modes in its topological phase. It is well-known that this system exhibits a topological–trivial superconducting phase transition. We demonstrate that high-harmonic spectroscopy is sensitive to the phase transition in presence of open boundary conditions due to the presence or absence of these edge modes. The population dynamics of the Majorana edge modes are different from the bulk modes, which is the underlying reason for the distinct harmonic profile of both the phases. On the contrary, in presence of periodic boundary conditions with only bulk modes, high-harmonic spectroscopy becomes insensitive to the phase transition with similar harmonic profiles in both phases.

## Introduction

Emission of radiation at prominently higher integer multiple frequencies of the incident laser frequency due to strong nonlinear interaction of intense laser fields with matter is known as high-harmonic generation (HHG)^1–3^. The emitted radiation encodes the information about laser-driven sub-cycle electron dynamics which forms the basis of HHG spectroscopy. In recent years, HHG in solids has become a method of choice to probe various aspects of condensed matter such as the observation of Bloch oscillations^4,5^, examining the dynamics of the defects in solids^6,7^, band structure tomography^8–10^, observation of the valley pseudospin^11,12,13^, imaging valence electrons^14,15^, realisation of petahertz currents in solids^16,17^, probing Berry phases^18,19^, and phase transitions driven by both light and topology^20–28^.

In the field of strongly correlated matter, topological superconductivity and associated phase transitions is presently one of the major topics^29,30^ as it is associated with the emergence of the Majorana fermion – particles that are their own antiparticles^31^. The notion of the Majorana fermion is also of great importance in nuclear and particle physics, apart from solid-state physics^32^. In a condensed matter context, Majorana fermions are emergent quasiparticle excitations that have been proposed as the physical basis to realize qubits that are robust against decoherence and fault-tolerant topological quantum computation^33–36^. In this work, we discuss the role of the Majorana fermions by probing the topological–trivial superconducting phase transition using HHG spectroscopy.

Experimental observation of Majorana fermions is an ongoing quest, and several experiments on semiconductor nanowires, magnetic chains and superconductors, using transport and electrical measurements with scanning tunneling spectroscopy and Coulomb-blockade spectroscopy, have been reported^37–44^. Topological superconductors are considered as a promising platform for Majorana fermions since the original proposal of Kitaev^45^. Thus for this study, we choose the paradigmatic one dimensional (1D) Kitaev chain of spinless fermions as a suitable model system. The many-body Hamiltonian for this 1D superconducting chain is

1 $$ {\mathcal {H}}=  -\mu \sum _{j=1}^{L}c_{j}^{\dagger }c_{j}-t_0\sum _{j=1}^{L}\left( c_{j+1}^{\dagger }c_{j} + c_{j}^{\dagger }c_{j+1} \right) +\Delta \sum _{j=1}^{L}\left( c_{j}c_{j+1}+c_{j+1}^{\dagger }c_{j}^{\dagger } \right) . $$

Here, $\mu$ is the onsite energy, $t_0$ is a nearest neighbour hopping term, and $\Delta$ is an order parameter (chosen to be purely real). The term with $\Delta$ is a superconducting pairing term in mean-field approximation, which creates or annihilates pairs of particles at neighbouring lattice sites. $c_j^\dagger$ ($c_j$) is fermionic creation (annihilation) operator at site *j* and *L* represents the number of lattice sites.

In the appropriate region of parameter space, the system described by Eq. (1) can host a topological superconductor that leads to the emergence of Majorana zero-energy edge modes (MZMs)^32,42,43^. The boundary of the phase transition from the topological to the trivial superconducting side depends on the critical values of $\mu$, $t_0$ and $\Delta$^29,32^. For example, for $\Delta = t_0$, the system is in the trivial phase when $\left| \mu /t_0\right| > 2$ in the limit $L\rightarrow \infty$. This is qualitatively similar to the limit $\left| \mu /t_0\right| \rightarrow \infty$. For $\left| \mu /t_0\right| < 2$, the system behaves as a topological superconductor with boundary MZMs in presence of edges. At $\left| \mu /t_0\right| = 2$, the gap closes which causes the boundary zero modes to hybridize and disappear^34^.

**Figure 1:**
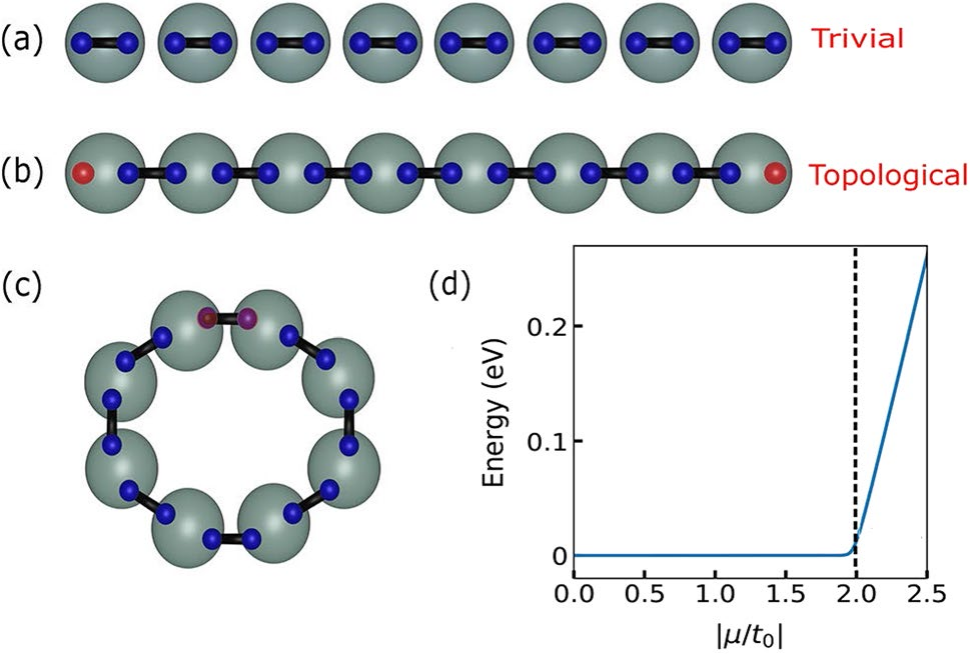
Notion of Majorana fermions in one dimensional chain. Two phases of the 1D superconducting Kitaev chain with open boundary: (**a**) the trivial phase has Majorana fermions (blue spheres) bound in pairs located on the same site of the physical lattice, represented by grey spheres. The other phase is (**b**) the topological phase, where Majorana fermions are bound in pairs located on the neighbouring sites leading to two unpaired Majorana fermions at both ends of the chain, represented by the red spheres. (**c**) The two unpaired Majorana at the ends of the chain again become bound in a pair when periodic or closed boundary condition is employed. (**d**) Lowest many-body excitation energy for $L = 128$ as a function of $|\mu /t_{0}|$.

To set the stage for the HHG study, we briefly recall this topological–trivial phase structure and its association with the MZMs. Following Ref.^45^, one rewrites Eq. (1) in terms of Majorana operators $c^\dagger _j = \frac{1}{2}(\gamma _{j,1} + i \gamma _{j,2})$ and $c_j = \frac{1}{2}(\gamma _{j,1} - i \gamma _{j,2})$ to arrive at ${\mathcal {H}} = \frac{i}{2} \Big [ -\mu \sum _{j=1}^{L} \gamma _{j,1} \gamma _{j,2} + (-t_0 + \Delta ) \sum _{j=1}^{L} \gamma _{j,1} \gamma _{j+1,2} + (t_0 + \Delta ) \sum _{j=1}^{L} \gamma _{j,2} \gamma _{j+1,1} \Big ]$ up to a constant. The system is in the trivial phase when two Majorana fermions are bound on the same site as sketched in Fig. 1a. This is clearly seen when $\mu$ is the dominant scale in ${\mathcal {H}}$ as expressed in terms of the Majorana operators. On the other hand, two unpaired Majorana fermions emerge at the boundary when Majorana fermions are bound in pairs on the neighbouring sites as sketched in Fig. 1b that corresponds with the system being in the topological superconducting phase. This is easiest to see for $t_0 = \Delta$ and $\mu =0$. More generally in the topological phase, it is understood that the two unpaired Majorana fermions (or MZMs) are exponentially localised at each physical boundary^45^. This notion of emergent unpaired Majorana fermions at the boundary or edge, expectedly, gets lost when closed (periodic) boundary condition (PBC) is employed as sketched in Fig. 1c. The topological–trivial transition can be seen by tracking the lowest many-body excitation energy as a function of $\left| \mu /t_0\right|$ for the open chain as shown in Fig. 1d. On the topological side, there is an effectively zero energy (fermionic) excitation which is composed from the unpaired Majorana modes localised at the opposite edges as in Fig. 1b, whereas on the trivial side, such zero energy modes are lost.

**Figure 2:**
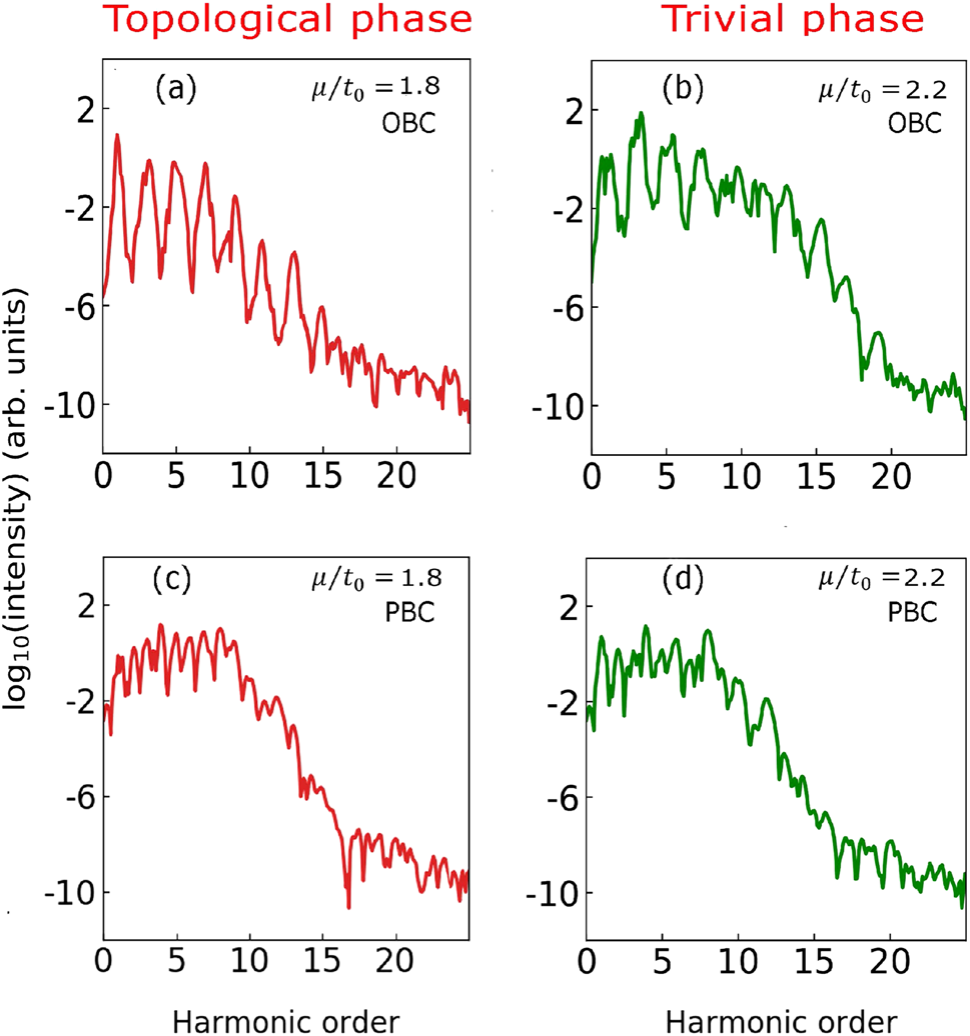
High-harmonic spectrum as a function of $\mu /t_{0}$ with different boundary conditions. High-harmonic spectra on the two side of the phase transition of the 1D superconducting Kitaev chain. The spectra for (**a**) the topological phase at $\mu /t_{0} = 1.8$, and (**b**) the trivial phase at $\mu /t_{0} = 2.2$ when the open boundary condition (OBC) is used. The spectra (**c**) and (**d**) are same as (**a**) and (**b**), respectively, with periodic boundary condition (PBC) imposed on the Kitaev chain. $L = 128$ is used while simulating the harmonic spectra. A linearly polarised laser pulse with a peak amplitude of 30 MV/m and 9.1 $\upmu$m wavelength having ten optical cycles with sine squared envelope is used to obtain the spectrum.

High-harmonic spectra for the 1D Kitaev chain with open boundary conditions (OBC) and PBC are presented in Fig. 2. The left and right panels in the figure correspond to topological and trivial phases, respectively, on either side of the transition at $\mu /t_0 = 2.0$. To observe the sensitivity of HHG spectroscopy on the two phases, we have chosen the values of $\mu /t_0$ close to transition point. A gradual decrease in the harmonic intensity up to 15th harmonics is visible when the system is in the topological phase at $\mu /t_0 = 1.8$ (see Fig. 2a).

**Figure 3:**
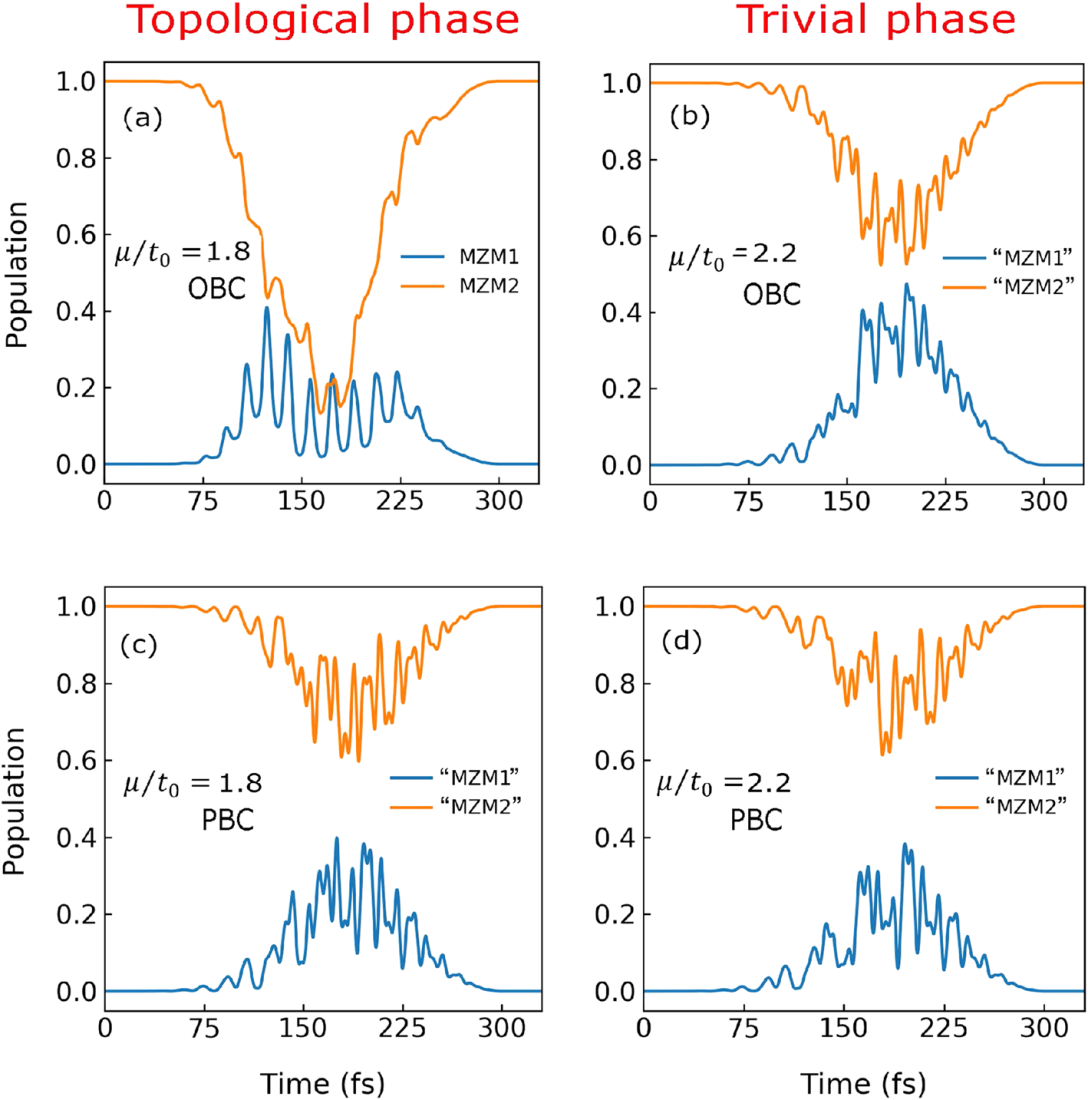
Population dynamics of Majorana zero modes in the presence of strong laser pulse. Population dynamics during the laser pulse for (**a**) doubly degenerate Majorana zero-energy modes (MZM1 and MZM2 in the legend) in the BdG spectrum on the topological side at $\mu /t_0 = 1.8$ and (**b**) the corresponding non-zero non-degenerate modes (which we have still called “MZM1” and “MZM2” to avoid extra notation) on the trivial side at $\mu /t_0 = 2.2$ with OBC. The population dynamics (**c**) and (**d**) are same as (**a**) and (**b**), respectively with PBC where none of the modes are at zero energy due to the absence of MZMs.

However, the spectrum for the trivial phase at $\mu /t_0 = 2.2$ is significantly different from the topological phase, as evident from the right panel of the figure (see Fig. 2b). In this case, the extent of the harmonics is relatively broad, ranging from 3rd to 15th order. The intensity of the harmonics in the plateau region is comparable, and the spectra extend up to the 17th harmonic. In both the phases, the harmonic spectra exhibit odd harmonics only as expected from the inversion symmetry present in the 1D system. Note that energy eigen-spectra are almost same at $\mu /t_0 = 1.8$ and 2.2 as evident from Fig. S1. The band gap at $\mu /t_0 = 1.8$ is $\sim$0.109 eV (if MZM states at zero energy are excluded), and at $\mu /t_0 = 2.2$ is $\sim$0.11 eV. In Fig. S7, we also present a series of harmonic spectra for different values of $\mu /t_0$ (= 1.6, 1.7, 1.8, 1.9, 2.1, 2.2, 2.3, 2.4) across the transition through which their differences on the two sides of the transition becomes evident.

**Figure 4:**
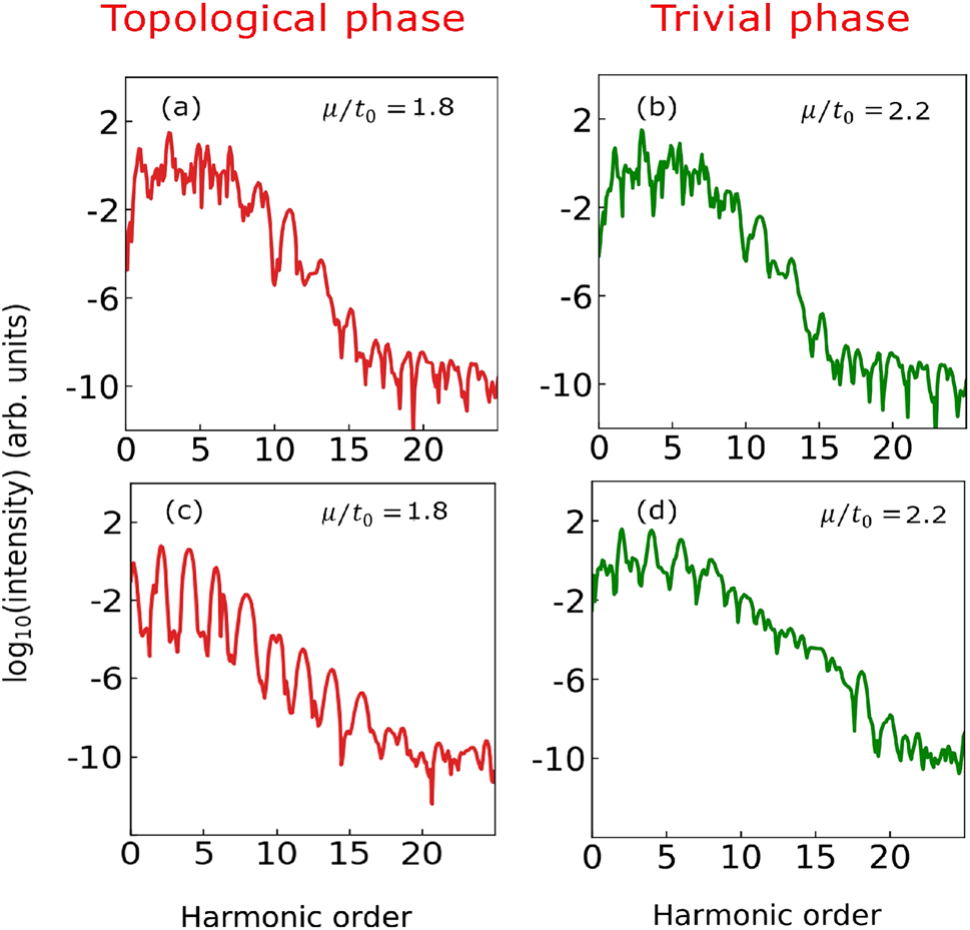
High-harmonic spectrum with and without Majorana zero modes of the 1D superconducting Kitaev chain with open boundary condition (OBC). Harmonic spectrum where for both occupied and unoccupied MZM states are not considered for (**a**) topological phase $\mu /t_{0} = 1.8$, and (**b**) trivial superconducting phase $\mu /t_{0} = 2.2$. Harmonic spectrum originating only from occupied MZM state for (**c**) topological phase $\mu /t_{0} = 1.8$, and (**d**) trivial superconducting phase $\mu /t_{0} = 2.2$. A linearly polarised laser pulse with a peak amplitude of 30 MV/m and 9.1 $\upmu$m wavelength having ten optical cycles with sine squared envelope is used to obtain the spectrum.

The situation becomes very different when PBC is imposed on the 1D Kitaev chain such that there are no boundaries. In this case, the harmonic spectra corresponding to $\mu /t_0 = 1.8$ and 2.2 are very similar as reflected from Fig. 2c,d. The small differences between both the spectra arise due to slight changes in the eigen-spectra at $\mu /t_0 = 1.8$ and 2.2. The analysis of Fig. 2 concludes that the harmonic spectra are very similar when PBC is employed, whereas markedly different for both the phases when OBC is employed. This indicates that the MZMs are playing a distinct role in the harmonic generation as seen by the contrast in the HHG spectra in both the phases with different boundary conditions. In the following, we will investigate how the MZMs are associated with the different harmonic spectra in the two phases.

To elucidate the role of the MZMs in the harmonic generation, population dynamics of the doubly degenerate MZMs in Bogoliubov-de Gennes (BdG) spectrum on the topological side is presented in Fig. 3. We recall that the degeneracy of the two zero modes is lifted in the trivial phase, whereby they lose their Majorana character. When compared to these corresponding non-degenerate and non-zero modes in the trivial phase, the population dynamics during the laser pulse on the topological side at $\mu /t_0 = 1.8$ shows that the degenerate Majorana states show drastically different behaviours (see Fig. 3a,b). This noticeable difference in population dynamics in both the phases owing to the topologically enforced zero mode character of the Majorana excitations or lack thereof can be correlated with the different harmonic spectra observed in both the phases as shown in Fig. 2.

In presence of PBC, when there are no MZMs present, the temporal evolution of the corresponding modes are quite similar at $\mu /t_0 = 1.8$ and 2.2 (see Fig. 3c,d). This similarity in their population evolution indicates that both the modes are contributing in the same way to the harmonic generation at $\mu /t_0 = 1.8$ and 2.2; this correlates with almost the same harmonic spectra for both the phases with PBC as shown in Fig. 2c,d in contrast to the above discussion on the previous case with OBC. Moreover, their behaviour is similar to the OBC case on the trivial side (Fig. 3b) when these modes have become part of the bulk and not localized at the boundary anymore. We recall again that the notion of the unpaired Majorana fermions exists only on the topological side with OBC which is in line with the preceding discussions. The present findings thus establish that the harmonic spectra and the population dynamics of the MZMs are different for both the phases for the 1D Kitaev chain with OBC. This concludes that high-harmonic spectroscopy is sensitive to the presence of the MZMs. The notion of unpaired Majorana fermion does not exist for the 1D Kitaev chain with PBC. As a result, the harmonic spectra and the population dynamics are similar for different values of $\mu /t_0$.

In order to highlight further the role of MZMs in the differences of the harmonic profiles in presence of OBC, we also show the harmonic spectra obtained by artificially suppressing the presence of MZMs in the eigen-spectra. The harmonic spectra at $\mu /t_0 = 1.8$ and 2.2 with OBC are almost same as evident from Fig. 4a,b. The minor difference in the spectra are originating from the minor difference in the eigen-spectra. Moreover, the harmonic spectra solely originating from one occupied MZM at $\mu /t_0 = 1.8$ and corresponding non-zero mode at $\mu /t_0 = 2.2$ is shown in Fig. 4c,d. As reflected from the figure, the spectra are drastically different from each other. Furthermore, the population dynamics of the highest occupied and lowest unoccupied eigen-states (representative of the bulk modes) corresponding to the spectra in Fig. 4a,b are similar as expected (see Fig. S8). On the other hand, the population dynamics corresponding to spectra in Fig. 4c,d are drastically different (see Fig. S8). Results presented in Fig. 4 unequivocally establish that the similarities and differences in the spectra under different situations are due to the presence or absence of the MZM modes. Also, the system continues to exhibit different harmonic profiles in the two phases (for OBC) for different laser intensities (see Figs. S3 and S4) and wavelengths (see Figs. S5 and S6).

We now briefly comment on the domain of validity for the observations made earlier. Keeping our choice of the laser parameters fixed, we find that a variation of $\sim 11\%$ in the $t_0=\Delta$ scale does not change our conclusions. Keeping the $t_0$ scale fixed, a variation of $~\sim \pm 8\%$ in $\Delta /t_0$ keeps conclusions unchanged as well. Beyond these variations, the laser parameters have to be tuned accordingly to find the suitable values. Thus, analogous simulations can be helpful in determining the appropriate laser characteristics when applying HHG to different physical systems. Finally, it is expected that the HHG obtained from the time evolution of the many-body Hamiltonian in Eq. (1) and from that of the BdG-recasted “one-particle” Hamiltonian should be the same in principle. We have confirmed this expectation by explicit numerical simulations (results not shown here), which is an independent check on the accuracy of our numerical time evolution procedures.

In summary, our work demonstrates that HHG spectroscopy is sensitive to the topological–trivial phase transition in 1D superconducting Kitaev chain. The extent of the harmonic plateau and energy cutoff in the trivial phase are distinguishable from the harmonic spectra in the topological phase. This distinguishability is further consolidated by various harmonic spectra for different intensities and wavelengths of driving laser as presented in supplementary material S1. The population dynamics of the two Majorana states and their contributions to the total harmonic spectra are very different for both the phases, which we established to be correlated with the differences in the HHG spectra of the two phases. This distinct signature may potentially point to a new probe of Majorana modes in solids. A physical explanation for the numerically observed differences in the population dynamics of MZMs and bulk modes that were shown to underlie the HHG spectra is desirable for better understanding. Finally, the feasibility of coupling existing realisations of topological superconductivity with potential MZMs to *intense* laser fields in the laboratory is an open issue^46^, and we hope our work stimulates attempts in this direction.

## Methods

To probe the topological–trivial phase transition by HHG spectroscopy, we imagine that the 1D Kitaev chain is exposed to an ultrashort, strong laser pulse. For simulating this process, it is useful to recast the Hamiltonian, given in Eq. (1), in the two-component Nambu-Bogoliubov-de Gennes (BdG) formalism^47^. The Hamiltonian of Eq. (1) can be written as ${\mathcal {H}} = \frac{1}{2}\;C^{\dagger }\;H_{\text {BdG}}\;C$ with $C = (c_1,...,...,c_L,c^{\dagger }_1,...,...,c^{\dagger }_L)^T$ as a column vector containing all fermionic creation and annihilation operations. The one-particle matrix elements contained in $H_{\text {BdG}}$ can be compactly written as

2 $$ H_{\text {BdG}}= -\mu \sigma _{z}\sum _{n}\left| n \right\rangle \left\langle n \right| - \sum _{n} [(t_{0} \sigma _z - i \Delta \sigma _y) \left| n \right\rangle \left\langle n+1 \right| + \text {h.c.}] $$

by using Pauli matrices $\sigma$. $H_{\text {BdG}}$ thus has the dimension of $2L \times 2L$, where $|n\rangle$ corresponds to the *n*-th site of the chain and $\text {h.c.}$ stands for the hermitian conjugate. Here, $\sigma$ operates on the particle and hole indices of the BdG formalism.

In presence of the laser pulse, the hopping term now acquires a time-dependent Peierls phase $\theta (t)$. The time derivative of $\theta (t)$ is directly proportional to the applied electric field as $E(t) = -\frac{1}{a_0}\frac{d\theta (t)}{dt} = -\frac{dA(t)}{dt}$ with $a_0$ as the lattice constant and *A*(*t*) as the time-dependent vector potential of the driving laser pulse. Here, we are thus assuming a spatially constant field due to the laser. This assumption corresponds to the limit when the wavelength of the laser is much larger than the 1D system under probe which will govern our choice of laser wavelength subsequently. Also, the value of $\Delta$ changes as a function of time in the presence of laser (see Fig. S2). To simulate the time-dependent changes in (real) $\Delta$, we have used the fact $\Delta (t) \propto \langle c_j c_{j+1} \rangle (t)$ for the pairing term as time progresses, e.g. see Ref.^48^. Time-dependent Schrodinger equation for the modes of the BdG-recasted Hamiltonian is numerically solved to simulate the high-harmonic spectrum along with time-evolving $\Delta (t)$ – i.e., at each (small) discrete time step, the BdG modes are first computed using the Hamiltonian parameters from the previous time step, and then the pairing term coefficient $\Delta$ is re-computed using these new set of BdG modes just computed to supply the Hamiltonian parameters for the next time step. One ensures the convergence of this procedure with respect to the size of the discretized time steps which simulates the continuous time evolution.

The high-harmonic power spectrum is obtained by the modulus square of the Fourier-transform of the total current $j(t) = \sum _k\left\langle \psi _k(t)|{\hat{J}}(t)|\psi _k(t) \right\rangle$ where

3 $$\begin{aligned} {\hat{J}}(t) = -ie a_{0} t_{0}\sigma _z \sum _{n} [\mathrm {e}^{-i\theta (t)} \left| n \right\rangle \left\langle n+1 \right| - \text {h.c.}], \end{aligned}$$

and $|\psi _k(t) \rangle$ is the time-propagated state at time *t*. Half of the states in the BdG spectrum make up the many-body ground state which is considered as the initial state for the time evolution in order to obtain the harmonic spectra. On the topological side, this corresponds to (lower) one of the two zero-energy modes in the BdG spectrum being occupied.

In this work, $t_{0}$ = 0.26 eV and $a_{0} \sim$ 0.4 nm are considered for the simulations. In what follows, we set $\Delta = t_{0}$ at the initial time instant to describe our main observations. A linearly polarised laser pulse of 9.1 $\upmu$m wavelength having ten optical cycles with sine squared envelope is used. The polarisation of the driving pulse is along the 1D Kitaev chain and has a peak amplitude of 30 MV/m. Choice of the laser parameters is motivated from Ref.^49^. Time-step of 0.1 atomic unit ($\sim$ 2.5 as) is used for time propagation.

## Supplementary Information

Supplementary Information.

## Data Availability

Data that support the plots within this paper and other findings of this study are available from the corresponding authors upon reasonable request.

## Code availability

Code that support the findings of this study are available from the corresponding authors on reasonable request.
