# Supplementary material for: Role of Majorana fermions in high-harmonic generation from Kitaev chain
Source: Sci Rep. 2022 Apr 25;12:6722. doi: 10.1038/s41598-022-10465-9 (PMC9038912; doi:10.1038/s41598-022-10465-9)
Supplement: Supplementary file 1 — Supplementary Information. [file 41598_2022_10465_MOESM1_ESM.pdf]

Supplementary Material: Role of Majorana Fermions in high-harmonic generation from Kitaev chain

Adhip Pattanayak[1], Sumiran Pujari[1], and Gopal Dixit[1]

[1] *Department of Physics, Indian Institute of Technology Bombay, Powai, Mumbai 400076, India*

(Dated: January 28, 2022)

Supplementary Note 1: Bogoliubov-de Gennes Energy Eigenspectrum.

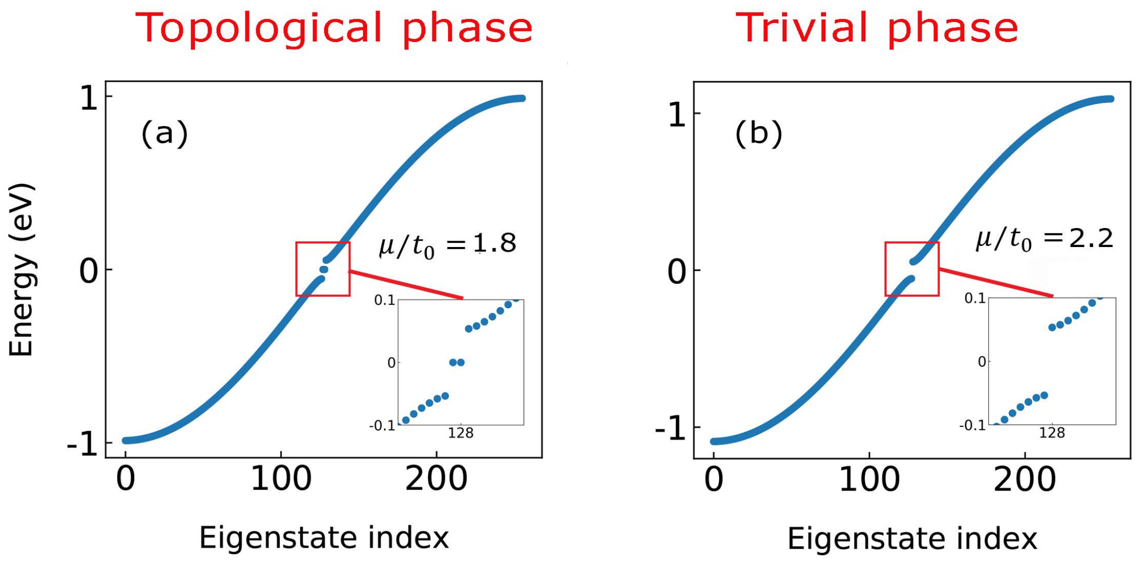

Supplementary Fig. S1. Bogoliubov-de Gennes Energy eigenspectrum of system in (a) topological phase $\mu/t_0 = 1.8$, and (b) trivial superconducting phase $\mu/t_0 = 2.2$.

Supplementary Note 2: Time-Variation in Δ

$\Delta(t)/t_0$ varies with time in the presence of strong laser field. The value of $\Delta(t)/t_0$ remains to its initial value approximately up to 3 optical cycles of the laser. A drop in the absolute value of $\Delta(t)/t_0$ is observed near the end of the third optical cycle. The value of $\Delta(t)/t_0$ decreases approximately to 7% at the end the laser pulse. This variation in Δ corresponds to a laser parameter with field amplitude 3×10^8 V/m, ten-optical cycles and frequency of 32.9 THz (0.136 eV) and *sine* squared envelope. We have performed similar numerical calculation for different set of laser parameters to get time-dependent $\Delta(t)$ for that particular set.

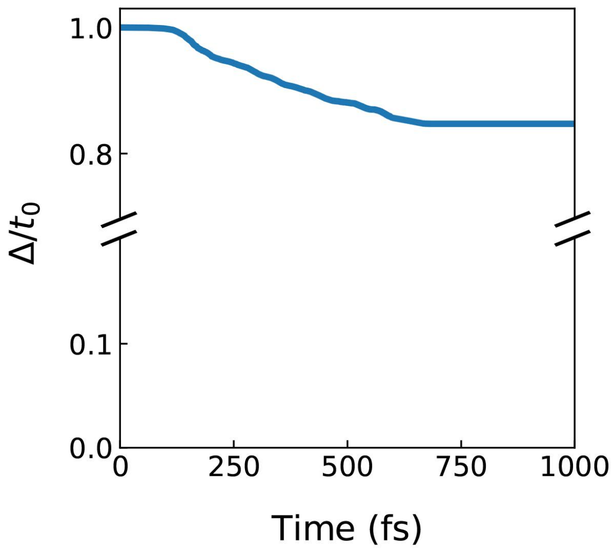

Supplementary Fig. S2. Temporal variation of Δ in the presence of the ten-optical cycles, *sine* squared envelope laser with field amplitude 3×10^8 V/m, and frequency of 32.9 THz (0.136 eV).

Supplementary Note 3: High-Harmonic Spectra with different laser intensities

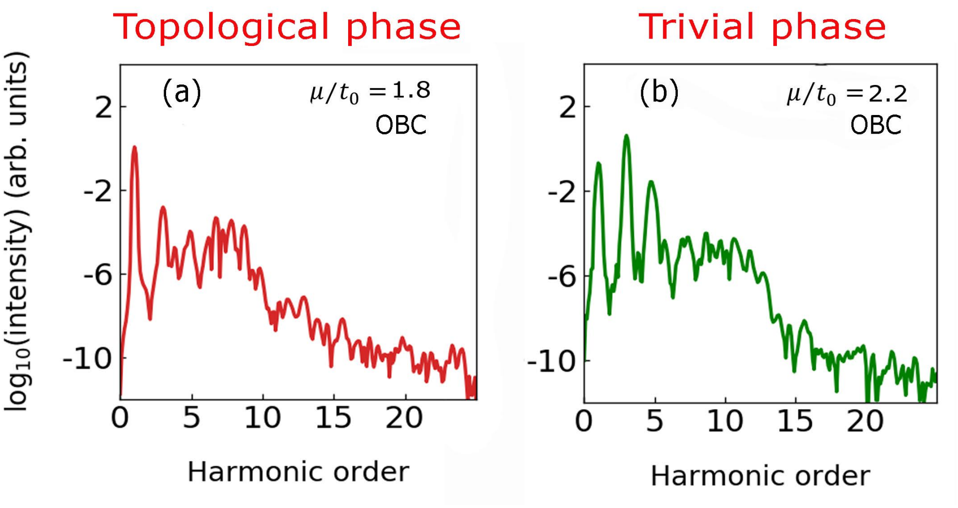

Supplementary Fig. S3. High-harmonic spectrum for (a) topological phase $\mu/t_0 = 1.8$, and (b) trivial superconducting phase $\mu/t_0 = 2.2$ of the 1D superconducting Kitaev chain with open boundary condition. The spectra correspond to a laser pulse with frequency 32.9 THz and amplitude 8×10^7 V/m.

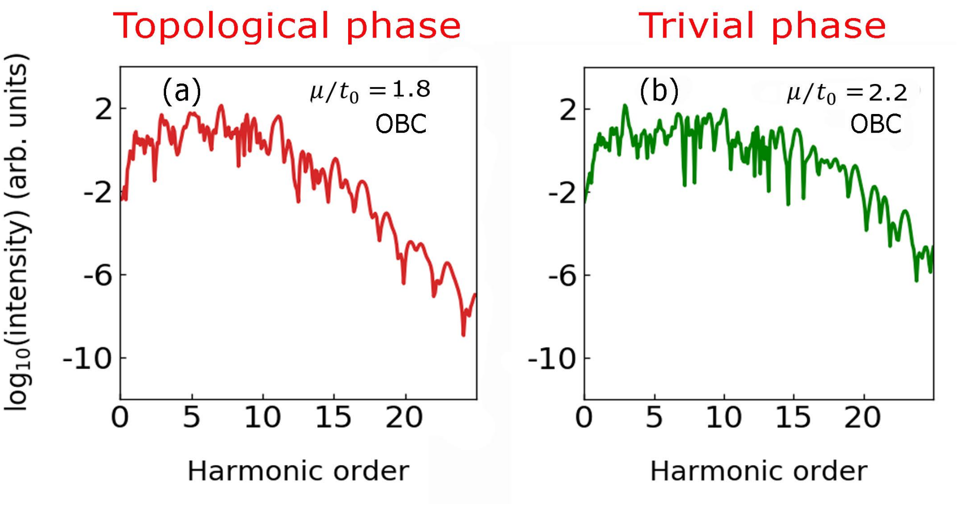

Supplementary Fig. S4. Same as in Fig. S3 except laser with amplitude 8×10^8 V/m.

Supplementary Note 4: High-Harmonic Spectra with different laser wavelengths

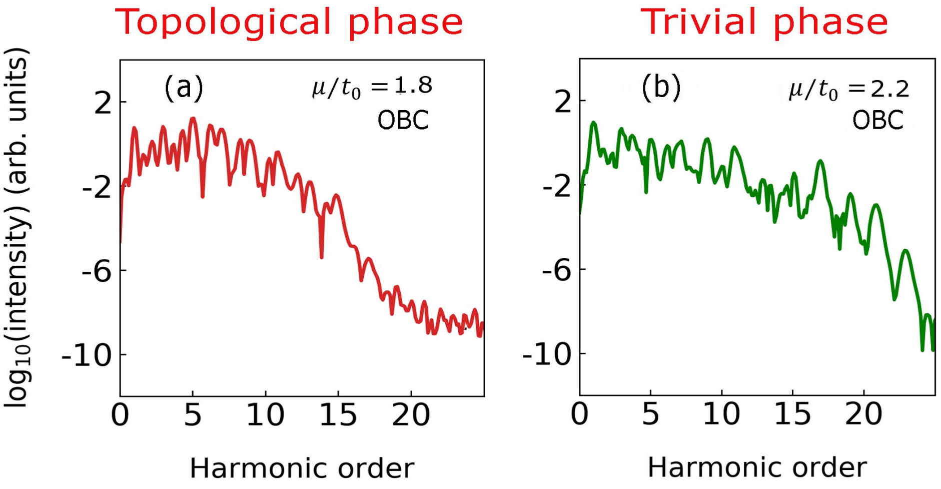

Supplementary Fig. S5. High-harmonic spectrum for (a) topological phase $\mu/t_0 = 1.8$, and (b) trivial superconducting phase $\mu/t_0 = 2.2$ of the 1D superconducting Kitaev chain with open boundary condition. The spectra correspond to a laser pulse with frequency 26.6 THz and amplitude 8×10^8 V/m.

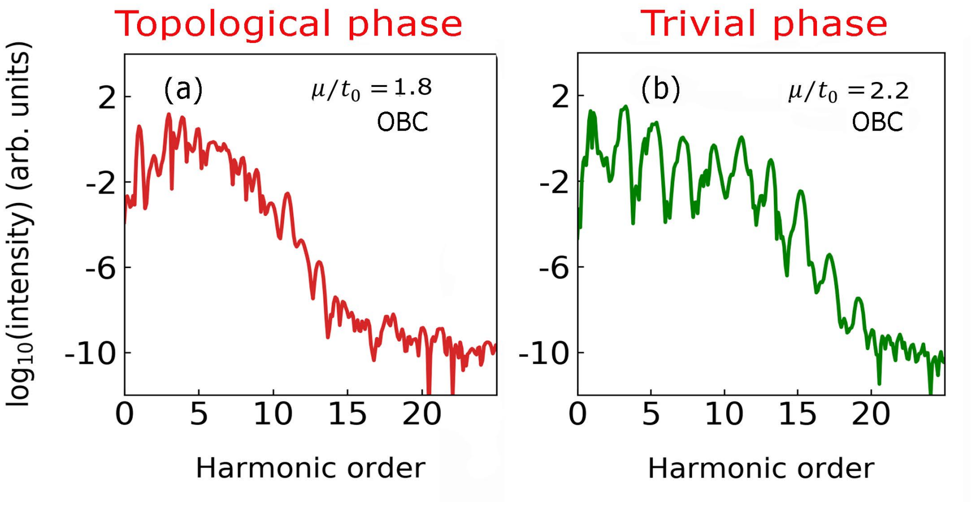

Supplementary Fig. S6. Same as in Fig. S5 except laser with frequency 36.2 THz.

Supplementary Note 3: High-Harmonic Spectra

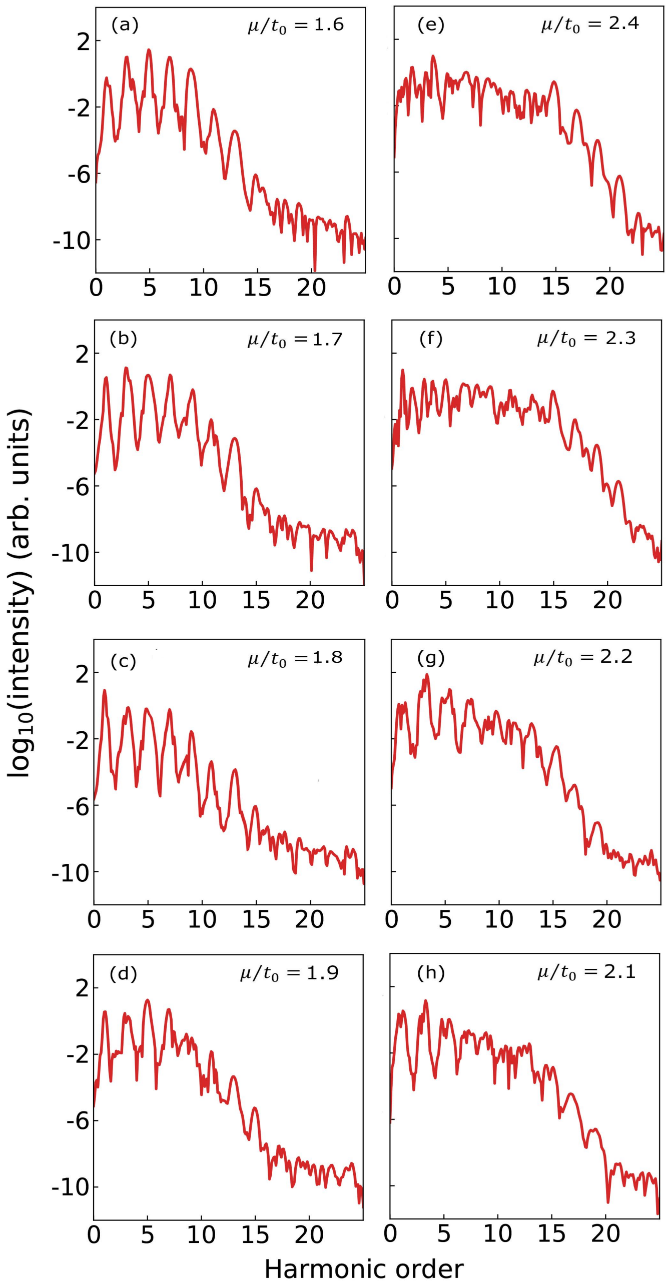

Supplementary Fig. S7. High-harmonic spectrum for different values of μ/t_0 across the topological–trivial superconducting phase transition in 1D superconducting Kitaev chain with open boundary condition. The spectra correspond to a laser pulse with frequency 32.9 THz and amplitude 8×10^7 V/m.

Supplementary Note 4: Population Dynamics

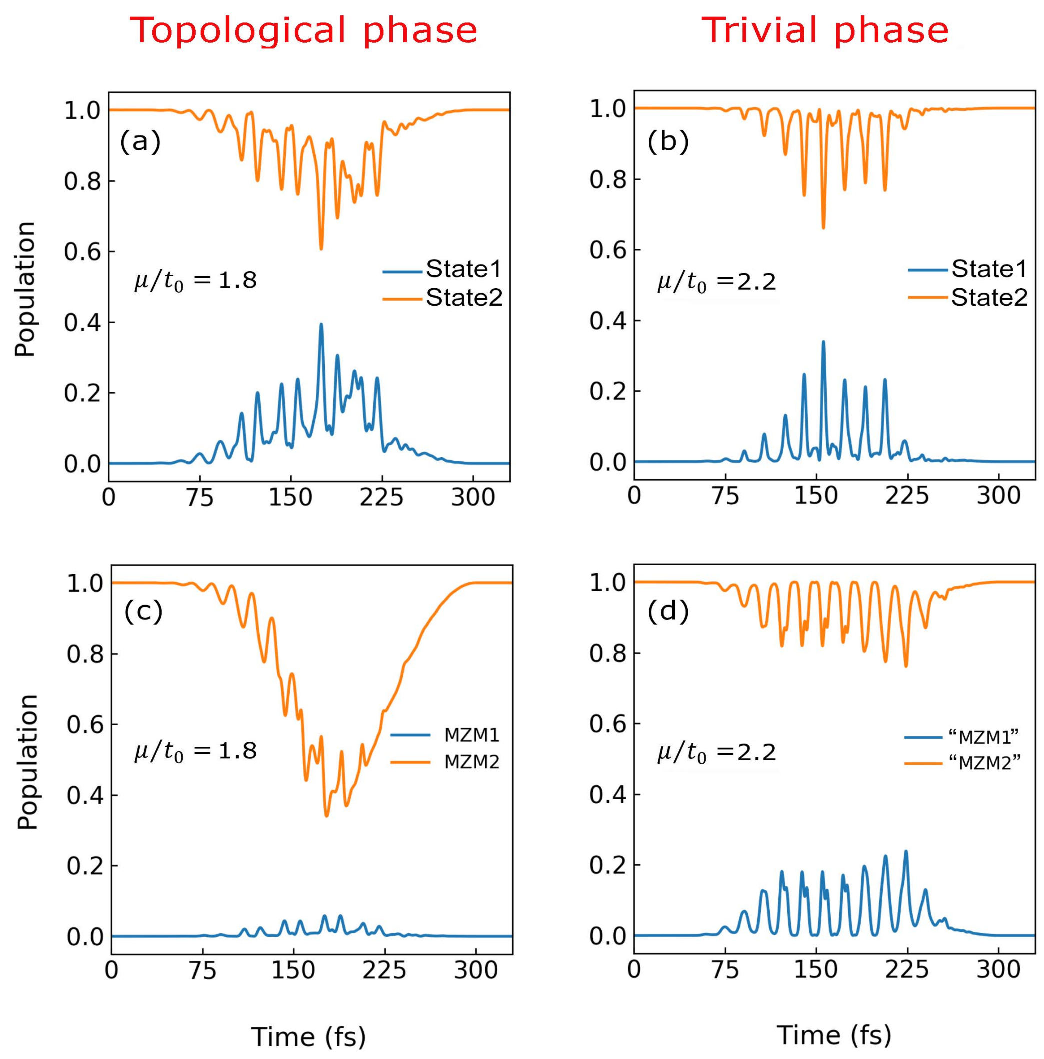

Supplementary Fig. S8. Population dynamics of the eigen-states corresponding to Fig. (4) in the main text. (a) and (b) correspond to the population dynamics of highest occupied and lowest unoccupied eigen-states of superconducting chain without Majorana zero modes. State2 and State1 represent highest occupied and lowest unoccupied eigen-states, respectively. (c) and (d) correspond to the population dynamics of superconducting chain having Majorana zero modes only. A linearly polarised laser pulse with a peak amplitude of 30 MV/m and 9.1 μm wavelength having ten optical cycles with sine squared envelope is used.
